# 281. Prevalence of Influenza Co-infection in a Real-world Cohort of COVID-19 Patients in the U.S

**DOI:** 10.1093/ofid/ofab466.483

**Published:** 2021-12-04

**Authors:** Devika Chawla, Xin Chen, Klaus Kuhlbusch, Kelly Zalocusky, Shemra Rizzo

**Affiliations:** 1 Genentech, Inc., South San Francisco, CA; 2 F. Hoffmann-La Roche, Basel, Basel-Stadt, Switzerland

## Abstract

**Background:**

Over 29 million people have been infected with COVID-19 in the U.S. alone. While COVID-19 carries serious morbidity and mortality, potential for co-infection with other respiratory infections remains unclear. We aimed to: (1) estimate co-infection prevalence of COVID-19 and influenza, and (2) compare demographics and clinical outcomes of co-infected patients to those of COVID-19 singly-infected patients using U.S. electronic health records (EHR).

**Methods:**

Patients in the Optum De-identified COVID-19 EHR database diagnosed with COVID-19 (lab-confirmed or ICD code) between February 2020 and January 2021 were eligible. Influenza co-infection was defined as an influenza diagnosis (lab-confirmed or ICD code) within ±10 days of COVID-19 diagnosis. We report co-infection prevalence for all COVID-19 patients and for a subset of hospitalized COVID-19 patients.

**Results:**

Among all COVID-19 patients (N = 549,532), 1,794 (0.3%) were co-infected with influenza. Among the hospitalized subset (N = 80,192), 242 (0.3%) were co-infected with influenza. In sensitivity analyses restricting to lab-confirmed influenza, co-infection prevalence was 0.1% overall and 0.2% among hospitalized patients. No meaningful differences were observed in baseline demographics between co-infected and singly-infected patients. Among hospitalized patients, univariate analysis suggested higher likelihood of invasive ventilation (12.8% vs. 9.8%; p=0.14), respiratory failure (56.2% vs. 46.6%, p< 0.01), and ICU stay (27.3% vs. 23.1%, p=0.13), but no meaningful difference in mortality (13.3% vs. 13.0%, p=0.97), for co-infected as compared to singly-infected COVID-19 patients.

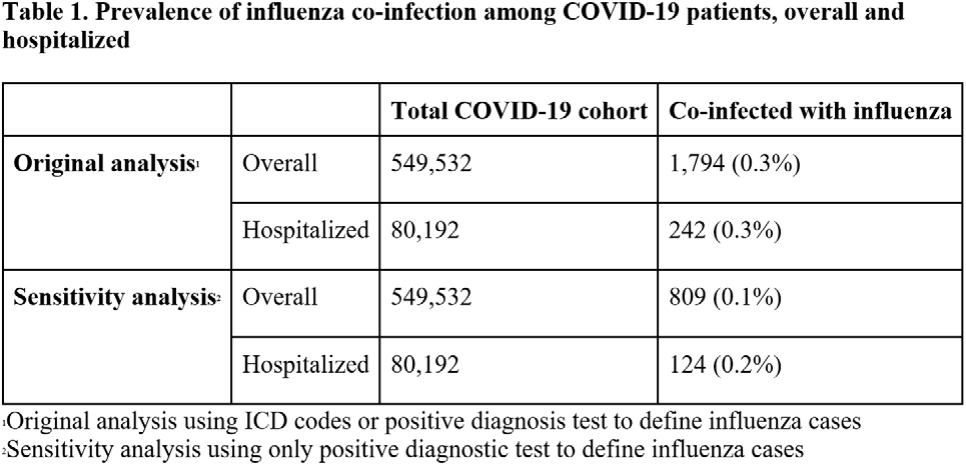

**Conclusion:**

In a real-world cohort, we observed a low proportion (0.3%) of COVID-19 patients co-infected with influenza. Co-infected patients had similar baseline characteristics but higher likelihood of hospitalization severity as compared to singly-infected COVID-19 patients. Limitations include low prevalence of circulating influenza and potential missing data bias.

**Disclosures:**

**Devika Chawla, PhD MSPH**, **F. Hoffmann-La Roche Ltd.** (Shareholder)**Genentech, Inc.** (Employee) **Xin Chen, PhD**, **F. Hoffmann-La Roche Ltd.** (Shareholder)**Genentech, Inc.** (Employee) **Klaus Kuhlbusch, PhD MD**, **F. Hoffmann-La Roche Ltd.** (Employee) **Kelly Zalocusky, PhD**, **F. Hoffmann-La Roche Ltd.** (Shareholder)**Genentech, Inc.** (Employee) **Shemra Rizzo, PhD**, **F. Hoffmann-La Roche Ltd.** (Shareholder)**Genentech, Inc.** (Employee)

